# From bottleneck to efficiency: Key areas for improving discharge management in healthcare – a qualitative study

**DOI:** 10.3205/000357

**Published:** 2026-06-01

**Authors:** Jann Niklas Vogel, Hanna Hilgenhof, Ivonne Honekamp, Chiara Kleinschmidt, Anne Petereit, Valerie Bühler, Stefan Schmidt

**Affiliations:** 1 University of Applied Sciences Neubrandenburg, Faculty of Health, Nursing, Management, Neubrandenburg, Germany; 2 University of Applied Science Stralsund, Faculty of Business Studies, Stralsund, Germany

**Keywords:** patient discharge, discharge management, transition management, care transition, care management, follow-up care

## Abstract

**Background:**

In Mecklenburg-Western Pomerania, multidisciplinary round tables bring together stakeholders from the healthcare sector to jointly develop and advance solutions for regional care challenges on a semi-annual basis. In a sparsely populated state with distinct structural and service-related difficulties, these collaborative formats play a key role in improving discharge management. Against this backdrop, the present study explores the main fields of action required to enhance discharge processes in rural areas of Mecklenburg-Western Pomerania. It aims to prioritise the key challenges, objectives, and measures that can contribute to a more efficient and patient-centred continuum of care following hospital discharge.

**Methods:**

Guideline-based group discussions were conducted at the round tables in Demmin, Pasewalk, Parchim and Ueckermünde and analysed using qualitative content analysis. To prioritise key topics and measures, a dialogue-based structural mapping method (Strukturlegetechnik) was applied. This approach enables the reconstruction of participants’ subjective theories through consensus-oriented discussions and visual mapping of conceptual relationships.

**Results:**

In four focus group discussions involving a total of 30 participants, three main areas for action were emphasised: the expansion of networks and binding coordination structures, the use of digital tools such as online platforms and telemedicine, and the development of tailored support services for vulnerable groups. In addition, prevention, health education, citizen participation and public relations were highlighted as essential cross-cutting tasks.

**Discussion & conclusion:**

To ensure sustainable healthcare provision in rural areas, it is essential to establish stable networks that meet on a regular basis and have reliable funding. The targeted expansion of telemedicine, digital communication platforms and capacity monitoring systems should be pursued to strengthen cross-sector collaboration and reduce gaps in service delivery.

## Zusammenfassung

**Hintergrund:**

In Mecklenburg-Vorpommern bestehen Runde Tische, an denen Akteur*innen des Gesundheitswesens multidisziplinär zusammenkommen, um halbjährlich gemeinsame Lösungsansätze für regionale Versorgungsprobleme zu entwickeln und deren Umsetzung zu begleiten. Gerade in einem Flächenland mit geringer Bevölkerungsdichte und spezifischen Versorgungsherausforderungen kommt diesen Strukturen eine zentrale Rolle für die Weiterentwicklung des Entlassmanagements zu. Vor diesem Hintergrund untersucht der Beitrag, welche Handlungsfelder für eine Verbesserung des Entlassmanagements in ländlichen Regionen Mecklenburg-Vorpommerns relevant sind, und zielt darauf ab, zentrale Herausforderungen, Zielsetzungen und Maßnahmen zu priorisieren, um eine effizientere und stärker patient*innenzentrierte Versorgung nach dem Krankenhausaufenthalt zu fördern.

**Methodik:**

Es wurden leitfadenbasierte Gruppendiskussionen an den Runden Tischen in Demmin, Pasewalk, Parchim und Ueckermünde durchgeführt, welche inhaltsanalytisch ausgewertet wurden. Zur Priorisierung zentraler Themen und Maßnahmen wurde die Strukturlegetechnik eingesetzt, ein dialogkonsensbasiertes Verfahren zur Rekonstruktion subjektiver Theorien.

**Ergebnisse:**

In vier Fokusgruppendiskussionen mit insgesamt 30 Teilnehmenden wurden vor allem drei Handlungsfelder betont: der Ausbau von Netzwerken und verbindlichen Koordinationsstrukturen, der Einsatz digitaler Instrumente (z.B. Portale, Telemedizin) sowie passgenaue Unterstützungsangebote für vulnerable Gruppen. Ergänzend wurden Prävention, Aufklärung, Bürgerbeteiligung und Öffentlichkeitsarbeit als zentrale Querschnittsaufgaben hervorgehoben.

**Diskussion & Schlussfolgerung:**

Für eine nachhaltige Gesundheitsversorgung in ländlichen Regionen sind stabile, regelmäßig tagende Netzwerke und eine gesicherte Finanzierung essenziell. Die Integration von Telemedizin, digitalen Kommunikationsplattformen und Kapazitätsmeldesystemen sollte gezielt vorangetrieben werden, um die sektorübergreifende Zusammenarbeit zu stärken und Versorgungslücken zu schließen.

## 1 Introduction

Discharge management (DM) comprises a set of coordinated measures designed to facilitate the transition of patients from hospital care to their home environment or to subsequent care services. Its overarching aim is to ensure seamless continuity of medical and nursing care, to prevent complications, and to enhance patients’ quality of life. Implementing DM in rural and structurally disadvantaged regions poses particular challenges. Healthcare services are often geographically dispersed, specialised facilities are only partially accessible, and workforce shortages further intensify existing constraints. Mecklenburg-Western Pomerania (MV) is especially affected by these conditions. With 68 inhabitants per square kilometre, it has the lowest population density in Germany [1]. Since 1990, the population has decreased by 15%, while the proportion of residents aged over 65 has doubled [2], [3]. These demographic trends place increasing pressure on local healthcare infrastructures and underscore the need for context-sensitive DM approaches. In response, four regional Round Tables (RTs) have been established in MV with the objective of fostering sustainable healthcare provision at the local level. Within these networks, regional stakeholders from healthcare, social work, politics, civil society, and public administration collaborate in a multidisciplinary manner to develop cross-sectoral strategies for acute and post-discharge care.

The collaborative research project NAHVERSORGT examines how DM is currently being implemented in MV and which structural factors shape this process. International evidence suggests that patients in rural regions face a higher risk of care discontinuities and adverse outcomes following hospital discharge [4], [5]. Building on this evidence, the present study seeks to address the following research questions:

1. How do key stakeholders in structurally weak, rural regions of MV prioritise challenges, objectives, and measures in DM?
2. Which proposals for improving DM are articulated, and which measures are perceived as particularly effective?

The study aims to generate a deeper understanding of the specific requirements of DM in rural contexts. Its findings are intended to provide practical insights and inform the further development of regionally adapted models of care.

## 2 Methods

A qualitative research design was chosen to capture the perspectives of participants involved in the RTs and to derive concrete recommendations for strengthening DM in rural regions. The group discussion format enables the collection and comparison of different perspectives and opinions on a specific topic [6], while also facilitating the joint development of solutions to complex challenges [7]. To structure the group discussions, a semi-standardised interview guide was developed based on the findings from the first focus group discussion (FGD) of the collaborative project [8] and the results of a preceding scoping review [9]. The guide was organised into four thematic dimensions (see Table 1 (Tab. 1)), each accompanied by open-ended questions. This approach ensured that all relevant topics were addressed during the discussions and that participants’ responses could evolve according to their individual priorities and perspectives.

In addition, the Structural Mapping Technique (SMT) was applied to identify priorities within the thematic areas and to explore regional differences between the RTs. The SMT enables participants to structure complex relationships both visually and cognitively. The resulting structure is shaped partly by formal relations such as “is a prerequisite for”, and partly by the spatial arrangement of concept cards, whereby cards positioned higher in the hierarchy are assigned greater importance [10]. The outcome is a structural map that makes reasoning patterns, thought processes, and conceptual linkages explicitly visible [10]. During the FGDs, participants prioritised the measures developed within their respective RT sessions and evaluated their relative significance. This process provided deeper insights into perceptions of key action areas and into regional variations among the RTs in MV. Data collection took place between 1 July 2025 and 22 July 2025 during regular RT meetings held in person. Each discussion lasted approximately 60 minutes. All sessions were moderated by a researcher experienced in qualitative interviewing to ensure methodological consistency and minimise procedural bias.

### Data analysis

The FGDs were transcribed, and the data were prepared and analysed using a category-oriented approach in a pseudonymised form. The method of structurally focused qualitative content analysis was applied [11] to organise and interpret the data material. This analytical approach allows for a combination of deductive and inductive reasoning. All steps of data analysis were conducted electronically using MAXQDA software (version 24.2.0). Ethical approval for the study was granted by the Ethics Committee of Neubrandenburg University of Applied Sciences (Ref. No.: HSNB/216/24).

## 3 Results

During the data collection period, group discussions were conducted across four RTs in MV with a total of 30 participants (n=18 female). Participants represented regional stakeholders from healthcare, social work, politics, civil society, and public administration. On average, the discussions lasted approximately 85 minutes. All contributions were included in the data analysis. The analysis resulted in the development of seven main categories (MCs) comprising a total of 111 differentiated subcategories at various levels (see Table 2 (Tab. 2)). Some categories served as organisational categories in the sense described by Kuckartz [11], structuring the overall category system but not necessarily being used directly for coding. The following section provides a detailed presentation of MC3 to MC6, which represent the core thematic areas of the study.

### MC3: Improving collaboration in discharge management

To optimise collaboration, participants primarily emphasised the need to improve interfaces and communication pathways between hospitals, physicians, nursing staff, social services, community coordinators and care support centres. Digital tools such as the Recare portal were described as a means of consolidating capacity requests and transmitting them collectively to potential aftercare facilities while also receiving feedback in return. The introduction of a pseudonymised local health record or the use of the electronic patient record was also mentioned as an option to enhance the flow of information. Regional reporting platforms and cluster models, similar to those established during the COVID-19 pandemic, were viewed as potentially useful for facilitating capacity notifications and needs assessments. Participants further stressed that data protection compliant communication structures were a key prerequisite. In addition, the need for stable personnel and financial support for coordinating roles, such as community managers or physician assistants, was highlighted:

*“[…] There is simply a lack of a manager to guide and coordinate the system. We have separate sectors, and I think there is great potential here that we could make better use of […]”* (H204, para. 7).

### MC4: Improving the health situation of vulnerable groups 

For vulnerable groups such as palliative care and dementia patients, or older people with multimorbidity, various measures were proposed. These included case management approaches and the deployment of health guides or community health nurses who provide support after discharge, assist with applications and pay attention to barriers within the home environment. Close collaboration with social services and care support centres was described as an important component of this ongoing support. Participants also suggested transitional and short-term housing options to bridge gaps in care provision. Examples included care apartments that can be temporarily shared with family members, cooperation with housing associations and hybrid models such as barrier-free holiday apartments with integrated care options. In this context, participants emphasised the importance of clear structural and architectural guidelines to ensure that housing providers and associations are aware of relevant standards:

*“Exactly, there needs to be something like a specification sheet […] for example, how wide the doors must be, how bathrooms should be designed, whether there should be separate cooking facilities, wheelchair accessibility, or whether a barrier-reduced or barrier-free design is sufficient.” *(S205, para. 68)

In addition, participants proposed the establishment of accessible model apartments to inform and raise awareness. Neighbourhood-oriented care concepts with local contact points, community spaces and the involvement of local networks were repeatedly highlighted as key strategies.

### MC5: Information dissemination and transparency in healthcare provision

Participants frequently described access to information about DM and subsequent care services as difficult or unclear. A recurring concern was therefore the establishment of digital platforms that consolidate regional services, contact persons and capacities, and that are regularly maintained:

*“My suggestion would be […] that such information should really be listed, that there is an overview of all services, and perhaps also online […] so that everyone has access to this overview and that it is continuously updated […].”* (O202, para. 38)

It was emphasised that such transparency would benefit not only patients themselves but also relatives and close contacts, who often provide support from a distance:

*“It is not only older people who look for these services, but often their relatives, who may live further away and check whether there is a support group for their father or mother and organise things from afar […].”* (O202, para. 40)

As complementary measures, participants proposed public outreach formats such as health fairs, themed days or community events that bring together the general public and professional stakeholders to promote direct exchange. Suitable venues mentioned included day care facilities, municipal buildings and community centres.

### MC6: Concrete recommendations for action

Within the fifth main category, Concrete recommendations for action, the SMT was applied to jointly identify priority measures with the participants. The weighting process illustrated which topics were perceived as particularly urgent and relevant. Overall, there was a strong emphasis on strengthening networking – both among healthcare actors within the region and across regional boundaries. This included the establishment of local physician networks, the closer integration of care support centres and the creation or reinforcement of coordinating roles. Another major focus was the development of digital infrastructures, including regional portals, databases of local service providers and online platforms that consolidate information and facilitate communication between providers. In several groups, the expansion of telemedicine was highlighted as a key strategy for addressing care gaps, particularly in remote areas. Prevention also emerged as a central area for action. Suggested measures included educational and awareness-raising activities on physical activity, nutrition and dementia, as well as training programmes for community multipliers to promote health literacy and preventive behaviour more widely: *“[…] we need to rethink prevention […] I think we should train more multipliers who embody prevention and bring it directly to people […].”* (H208, para. 106) The measures identified through the SMT can be grouped into several overarching thematic fields. Table 3 (Tab. 3) presents a location-independent overview of the discussed approaches, ordered by their frequency and relevance in the group discussions. Participants repeatedly stressed the importance of rethinking prevention and embedding it more firmly in everyday life to enhance acceptance. Across all FGDs, participants proposed conducting needs assessments to guide targeted service development – for example in respite care, emergency services and home adaptation. As a complementary measure, participatory citizen formats such as a “wish box” survey were mentioned. While participants across regions shared many priorities, differences in emphasis were also evident. Some RTs focused primarily on organisational structures and binding cooperation frameworks, whereas others placed greater emphasis on meeting spaces, educational activities and the expansion of digital tools such as telemedicine. Across all RTs, participants underlined the need for a broader societal shift towards preventive thinking as a long-term goal. Information and counselling services should become accessible earlier and more routinely in everyday life: *“Health literacy and preventive education should start as early as kindergarten and be sustained throughout life.”* (H207, para. 102) Participants also pointed to the importance of reducing existing prejudices towards care support centres and raising their profile as trusted points of contact:

*“Care support centres should play a central role. They provide accurate information and real support, but they are still not well known among the public […] they are truly competent and even visit people at home when needed […].”* (H207, para. 95)

Prevention was thus not regarded as a secondary activity, but as an integral, cross-cutting responsibility that can make a substantial contribution to strengthening regional healthcare systems.

## 4 Discussion

The findings from the FGDs not only confirm the central challenges and proposed solutions identified in the scoping review but also expand on them by incorporating specific perspectives on the unique structural and social conditions of rural regions in MV. The following section discusses these findings in relation to MC2 to MC5.

### MC2: Improving collaboration in discharge management

The results indicate that one of the key areas for action lies in optimising internal collaboration between hospitals, general practitioners, nursing staff, social services, community coordinators and care support centres. The main focus is on improving coordination within professional networks to prevent disruptions in the continuum of care. Participants particularly highlighted digital tools such as the electronic patient record and regional capacity reporting systems, which can help structure information flows between healthcare providers. Research projects in Germany have shown that such platforms can demonstrably improve communication, provided that they are used in a standardised manner [12] and are technically as well as terminologically compatible [13]. In addition, participants emphasised the importance of ensuring adequate staffing and sustainable funding for coordinating roles. The literature similarly highlights that clearly defined responsibilities and mandates are crucial for the success of such positions [14], [15]. Insights from German model projects such as the AGnES concept (Arztentlastende, Gemeindenahe, E-Health-gestützte Systemische Intervention – “physician-relieving, community-based, eHealth-supported systemic intervention”) further demonstrate that the structured delegation of medical tasks to qualified non-physician professionals can enhance the efficient use of medical resources and strengthen collaboration within regional healthcare networks [16], [17]. These approaches reveal the potential of innovative forms of cooperation but also underline that their success depends on clearly defined responsibilities and sustainable financing mechanisms. Taken together, the findings suggest that effective collaboration in discharge management cannot be achieved through digital tools alone. It requires the interplay of robust technical infrastructures, binding role definitions and reliable financial frameworks to ensure continuity and quality of care across sectors.

### MC3: Improving the health situation of vulnerable groups

The FGDs highlighted that vulnerable groups, such as older patients with multimorbidity, require specific and ongoing support. Participants identified case management, health navigator roles and community health nurses as suitable approaches to provide patients and their relatives with cross-sectoral contact points within the healthcare system. Comparable models have already been tested in Germany, for instance the “Pathfinders” within the TIGER project [18], [19] or various case management programmes [20], [21]. These examples demonstrate that such roles can improve orientation and navigation within the care system. However, participants and the literature alike noted that the sustainable funding and integration of these roles into existing structures remain unresolved challenges. Furthermore, participants emphasised the importance of transitional and short-term housing models, accessible demonstration apartments and cooperative housing arrangements designed to bridge care gaps after discharge and to prevent rehospitalisation. The Innovation Fund project StatAMed provides an illustrative example of how such cross-sectoral models can be implemented [22]. At the same time, the findings underscore the need for systematic evaluation and scaling of these approaches to ensure their long-term effectiveness and sustainability.

### MC4: Information dissemination and transparency in healthcare provision

Our findings indicate that a lack of transparency and the limited accessibility of information within DM represent key challenges in regional healthcare provision in MV. Digital platforms such as regional health portals, electronic patient records or aftercare databases were described by participants as promising innovations, as they consolidate information and facilitate faster navigation through the care process. In the literature, such tools are considered crucial for improving cross-sectoral information flows and ensuring continuity of care [23]. However, the effectiveness of these systems depends heavily on standardisation, interoperability and regular maintenance. Moreover, while digital transparency tools hold considerable potential for innovation, they also carry the risk of exacerbating existing inequalities: population groups lacking digital skills or access may be excluded. Against this backdrop, analogue formats such as local health fairs, community information days and outreach events remain highly relevant. A critical shortcoming is that these formats are often implemented sporadically and without systematic coordination. A promising way forward would therefore be to better integrate digital and analogue information channels and embed them more firmly within regional healthcare structures.

### MC5: Concrete recommendations for action

The prioritisation of themes within the SMT process showed that structural and organisational measures were considered particularly urgent by the participants. A key concern was to strengthen the formal structure and accountability of the RTs, including clearly defined goals, the allocation of responsibilities and regular meetings. The literature consistently highlights that these factors are critical to the success of interdisciplinary collaboration. Networks can only function effectively and sustainably when roles are clearly defined, communication channels are well established and organisational frameworks are supported at an institutional level [24], [25]. In practice, however, shortcomings in these areas often prevent promising collaborative initiatives from developing beyond the pilot stage. Participants also identified the expansion of digital infrastructure and the wider use of telemedicine as priority measures. Evidence shows that such technologies can improve access to care and speed up the exchange of information, particularly in regions with limited resources [26]. Participants further emphasised the importance of strengthening prevention and health literacy, including training for multipliers and targeted awareness campaigns, reflecting current national initiatives and programmes [27], [28]. Local assessments of existing services and needs, as well as participatory citizen formats such as “wish box” surveys, were described as essential first steps: *“But I think a kind of market analysis – identifying what is missing and what is already working – would definitely be very important.”* (S202, para. 243) These measures were seen as the foundation for more targeted planning and action. They also highlighted that identified needs can vary considerably between regions, underlining the importance of tailoring implementation strategies to local contexts.

### Limitations

This study was guided by Steinke’s core criteria for qualitative research [29]. Intersubjective transparency was ensured through detailed documentation of the analytical process in MAXQDA, independent content analysis by two researchers and the use of a clearly codified procedure. Empirical grounding [29] was achieved through a combined deductive and inductive approach, drawing on insights from the first FGD and the preceding scoping review. Several limitations should be acknowledged. The sample included only participants from the RTs in MV, which means the findings are shaped by the specific structural and social conditions of a rural and economically weaker region. Consequently, their transferability to other contexts is limited. Moreover, certain perspectives particularly those of patients were not represented. Additional FGDs involving further professional groups and affected individuals could therefore yield valuable complementary insights. FGDs may also be influenced by group dynamics and socially desirable responses [30]. The use of the SMT represents an innovative and distinctive element of this study. While the thematic structuring is partly subjective and complex interrelations can only be captured to a limited extent, the method nevertheless enables a systematic prioritisation and hierarchical organisation of topics across multiple sites. In combination with the FGDs, the SMT provided practical, comparable findings that directly supported the study’s objectives.

## 5 Conclusion

The findings of this study indicate that lasting improvements in DM within rural regions need to be pursued on three interconnected levels: by establishing stable and trustworthy networks, developing digital capacity and information systems, and expanding telemedicine infrastructure. The results also show that community health nurse models and flexible transitional or short-term care options can play a particularly valuable role in ensuring continuity and security of care for vulnerable groups. Preventive education initiatives and participatory engagement formats should be regarded not as supplementary measures but as essential components of a forward-looking care strategy. Beyond generating practical priorities for action, this study also contributes to a conceptual understanding of how DM in structurally weaker rural settings can be systematically advanced. Further research is needed to evaluate the implementation and effectiveness of the prioritised measures and to assess their transferability to other rural regions.

## Abbreviations

- DM: Discharge management
- FGD: Focus group discussion
- MV: Mecklenburg-Western Pomerania
- MC: Main category
- RT: Round Table
- SMT: Structural Mapping Technique

## Notes

### Acknowledgements

The authors would like to thank all participants of the Round Tables for their voluntary engagement and valuable contributions to the group discussions.

### Funding

This study forms part of the collaborative project “NAHVERSORGT – Na©hversorgt in der Region”. The project on which this publication is based was funded by the Innovation Committee of the Federal Joint Committee (G-BA) under grant number 01VSF23038.

### Competing interests

The authors declare that they have no competing interests.

## Figures and Tables

**Table 1:**
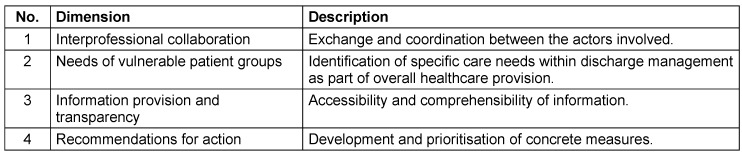
Dimensions of the focus group discussion guide

**Table 2:**
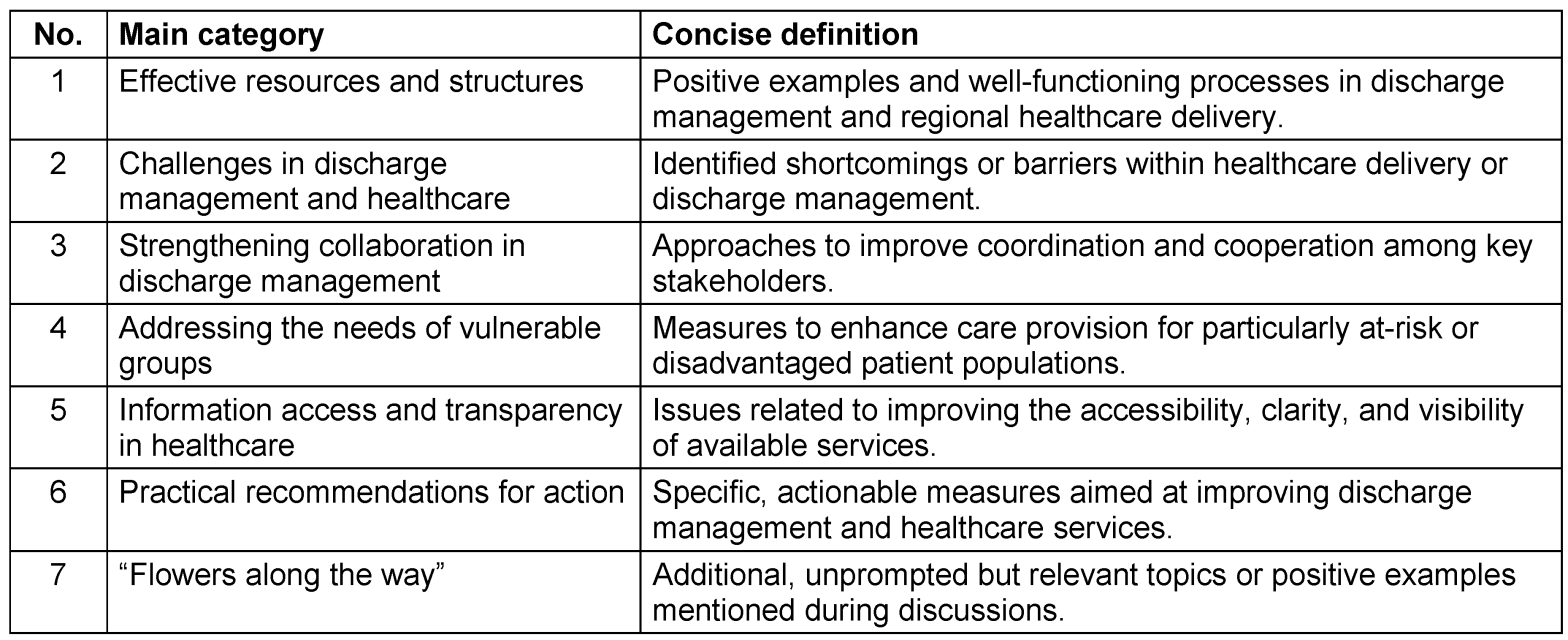
Overview of main categories with concise definitions

**Table 3:**
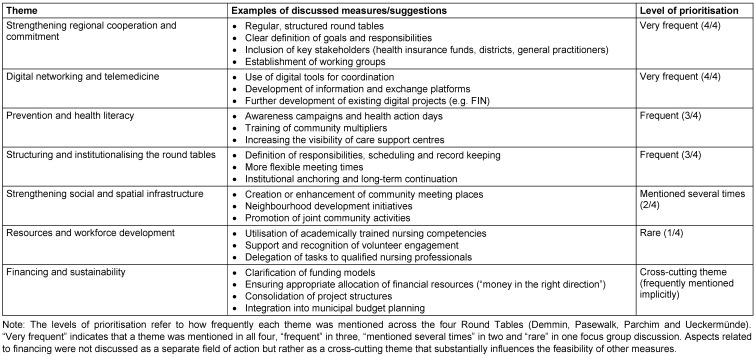
Overview of discussed themes and measures by level of prioritisation
